# A two-and-a-half-year-old breastfed toddler presenting with anemia: a case report

**DOI:** 10.1186/1756-0500-7-917

**Published:** 2014-12-16

**Authors:** Fabian Bock, Katrin Borucki, Peter Vorwerk, Ronald Biemann, Berend Isermann

**Affiliations:** Department of Clinical Chemistry and Pathobiochemistry, Otto-von-Guericke-University, Leipziger Straße 44, 39120 Magdeburg, Germany; Department of Pediatrics, Otto-von-Guericke-University, Leipziger Straße 44, 39120 Magdeburg, Germany; Department of Internal Medicine I and Clinical Chemistry, University of Heidelberg, INF 410, 69120 Heidelberg, Germany

**Keywords:** Anemia, Breastfeeding, Iron deficiency

## Abstract

**Background:**

Anemia is a common presentation in children but the differential diagnosis of iron deficiency and β-thalassemia remains a diagnostic challenge. Red blood cell indices have been shown to perform weakly in such scenarios. One potential cause is breastfeeding, but the evidence for unusually prolonged exclusive breastfeeding as a cause of iron deficiency anemia in older (>2 years) toddlers is sparse and the association of breastfeeding with iron deficiency in this age group of older toddlers is not unequivocally established. In this case we describe an unusual cause of nutritional iron deficiency anemia in the age group of 2–3 years.

**Case presentation:**

We describe a two-and-a-half-year-old Turkish boy who presented to our outpatient clinic with recurrent diarrhea and anemia. The patient was febrile (99.1°F) with pale skin and signs of mild dehydration. A reduced nutritional status with a weight of 11.5 kg between the 3rd and 10th percentile was noted. Nutritional evaluation revealed that the boy was still exclusively breastfed with more than 6 times breastfeedings per day. Iron supplementation ameliorated the anemia and reduced hypochromic red blood cells.

**Conclusion:**

The case demonstrates that unusually prolonged (longer than two years) exclusive breastfeeding is a potential cause of iron deficiency anemia in older toddlers. We discuss a simple combination of laboratory tests with ferritin and red cell distribution width that together with a nutritional evaluation provide a quick diagnosis and show that even at such an advanced stage of nutritional iron deficiency oral iron supplementation is an effective treatment.

## Background

Anemia is a common phenomenon in children and includes challenging differential diagnoses, such as infectious causes and anemia associated with thalassemia. However, the most common cause is iron deficiency [1]. In the US approximately 9% of all 1-3-year old infants have iron deficiency as a result of iron-deficient nutrition [2, 3]. This is of high importance as it is clinically established that iron deficiency has significant impact on cognitive and neuro motor development [2, 4].

Clinical manifestation of iron-deficiency anemia (IDA) is frequently absent or late in children and hemoglobin (with a cut off < 11 g/dl) has a poor sensitivity of only ~23% for iron deficiency in small children [5, 6]. Several indices, combining Hb (Hemoglobin), MCV (mean corpuscular volume) and RBC (red blood cell) count, such as the Mentzer or England and Fraser index, have been proposed and tested for the differential diagnosis of IDA and β-thalassemia [7, 8]. Unfortunately these red blood cell indices perform poorly in this scenario [9]. There are other parameters that are highly effective in screening for iron deficiency in small children, such as a reticulocyte hemoglobin content (Ret-Hb) of < 26 pg [10]. Several studies, however, suggest that Ret-Hb may not discriminate well between iron deficiency and β-thalassemia and that Ret-Hb should hence be used with caution in populations with a high prevalence of β-thalassemia, such as the patients with Mediterranean ancestry [11, 12]. Hence, alternative diagnostic approaches including a detailed nutritional history and a suitable combination of laboratory parameters are needed.

After the correct diagnosis of IDA it remains a challenge to identify the underlying cause. In infants nutritional iron deficiency, e.g. due to prolonged (>6 months) breastfeeding, is frequent. Prolonged exclusive breastfeeding (EBF) has been associated with IDA at 9 months of age and for low birth weight infants at 6 months of age [13, 14]. Accordingly, the current American Academy for Pediatrics (AAP) guidelines state that exclusively breastfed term infants should receive iron supplementation starting at 4 months of age [2]. However, the clinical consequences of exclusive breastfeeding are not unequivocally established. A recent analysis has shown a non-significant trend for the correlation of breastfeeding time and IDA and contradicting data exists showing no effect of prolonged EBF on iron status at 24 months of age [15, 16]. In addition, the WHO (World Health Organization) recommends exclusive breastfeeding for 6 months but states that breastfeeding may be continued for up to two years and beyond if complementary food is provided. In infants with prolonged EBF, iron supplementation significantly improved iron stores and prevented IDA at 9 months of age [17–19]. However, the effects in these studies were often temporary and – not unexpected – in cohorts where IDA prevalence was already low no effect of iron supplementation was observed at 9 months [17, 19]. Hence, whether, when and to which extend iron ought to be supplemented in these cases remains ill-defined and IDA secondary to prolonged EBF in older toddlers (>2 years) requires further evaluation. The current report discusses these issues in a case of an older toddler with unusually prolonged breastfeeding presenting with anemia.

## Case presentation

**Figure 1:**
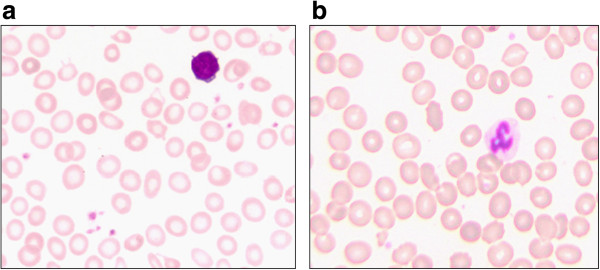
**a,b: Peripheral blood smear from a two-and-a-half-year old breast-fed patient.** The blood smear is showing hypochromic red blood cells before therapy **(a)**. Two months after therapy a decrease in hypochromic red blood cells was noted **(b)**.

We describe a 2.5 year old Turkish boy who presented to our outpatient clinic with recurrent diarrhea for the last 3 weeks following a family vacation. Diarrhea had a frequency of 10 per day and was accompanied by vomiting. The patient was mildly febrile (99.1°F) with pale skin and signs of mild dehydration with reduced skin turgor but quick recapillarization. Furthermore, a reduced nutritional status with a weight of 11.5 kg between the 3rd and 10th percentile and carious dentition were apparent. No other maladies, allergies, or use of medication were reported. Laboratory analysis revealed moderate-to-severe anemia: decreased Hb with 7.1 g/dl (10.8-12.7), MCV of 48 fl (73–101), MCH (mean corpuscular hemoglobin) of 0.71 fmol.(1.40-2.00), increased RBC count of 6.19 × 10^6^ cells/μl (3.70-5.30) and increased red cell distribution width (RDW) of 20.3% (11.5-14.5), thrombocytosis of 754,000 cells/µl (140–360) and increased reticulocyte count of 316 × 10^9^ cells/l (68–108) with reduced reticulocyte hemoglobin of 1.04 fmol (1.74-2.17) (Table 1). An increase of hypochromic RBCs (90%; Figure 1a), low values for serum-iron of 2 μM (5.7-18.6) and ferritin of 2 ng/ml (6–67) but normal CRP (C-reactive protein) (0.9 mg/l; <5), bilirubin (2.8 μM; <32) and haptoglobin (1.96 g/l; 0.30-2.00) were detected (Table 1). Fecal occult blood test was negative and no pathogenic protozoa, helminthes, shigella, yersinia, adeno-, noro-, rota-, astroviruses were found in coproculture. The initially suspected diagnosis of thalassemia was ruled out by a normal hemoglobin electrophoresis with 96.6% HbA1 (>96.3), 2.7% HbA2 (<3.2) and 0.4% HbF (<0.5). According to the information provided by the parents the patient’s sole source of nutrition was breast milk with a frequency of more than 6 breastfeds a day. While we cannot entirely rule out that the boy may have had solid supplements every now and then, potentially from other sources, it was evident from the history that the boy did not receive any food supplements on a regular basis. Given the nutritional status and the absence of enhanced hemolysis parameters the hypochromic microcytic anemia was attributed to nutritional iron deficiency. The parents were educated about the risks of under- and malnutrition, about the importance of a healthy diet, taking the current WHO recommendations into account [20] and about reducing the number of breastfeeds. Upon follow up visit the parents reported that the diet had been adopted accordingly. In addition, oral iron substitution was initiated. The Hb ameliorated (10.15 g/dl), hypochromic RBCs decreased to 15.7% and ferritin normalized (20.9 ng/ml) 2 months after discharge (Figure 1b).

**Table 1:** **Selected laboratory test results of the patient at the time of initial presentation**

Parameter (Unit)	Value	Reference range
Hemoglobin (g/l)	7.1	10.8-12.7
MCV (f)	48	73-101
MCH (fmol)	0.71	1.40-2.00
RBC count (10^6^ cells/μl)	6.19	3.70-5.30
WBC count (cells/nl)	7.6	5.0-12.0
Platelets (10^3^ cells/μl)	754	140-360
Reticulocyte count (10^9^ cells/l)	316	68-108
Retikulocyte hemoglobin (fmol)	1.04	1.74-2.17
Hypochromic RBCs (%)	90	<5
Ferritin (ng/ml)	2	6-67
RDW (%)	20.3	11.5-14.5
CRP (mg/l)	0.9	<5
Iron (μM)	2.0	5.7-18.6
Transferrin (g/l)	2.86	2.0-3.6
Transferrin-Saturation (%)	2.8	7-44
Haptoglobin (g/l)	1.96	0.30-2.00
Bilirubin (μM)	2.8	<32

## Discussion

The differential diagnosis of iron deficiency anemia (IDA) and anemia associated with thalassemia remains a challenge as RBC indices perform weakly in these scenarios [9]. Ferritin is a helpful parameter to distinguish IDA from thalassemia in patients with microcytic anemia [21–23]. Since ferritin does not always accurately reflect the iron stores (e.g. in acute infection) other parameters need to be considered. Parameters potentially to be used together with ferritin for the diagnosis of iron deficiency are the transferring saturation (using a cut-off of < 10%) and erythrocyte protoporphyrin (cut off > 1.42 μM) [6]. However, these parameters have limitations, too. Firstly, the transferrin saturation (cut off < 10%) has at best a sensitivity and specificity for iron deficiency similar to that of ferritin (cut off < 12 ng/ml) and hence does not provide an advantage [22, 24, 25]. Indeed, a reduction of the transferrin saturation has been proposed to be indicative of iron deficiency only in conjunction with manifest anemia [26]. Secondly, determining the transferrin saturation requires laboratory analyses of two parameters (iron and transferrin) and is hence more expensive. Lastly, erythrocyte protoporhyrin may be a sensitive indicator of iron deficiency but rates of erythropoiesis vary among children and it is also elevated in β-thalassemia questioning its usefulness in the differential diagnosis in our case [27].

The combination of ferritin with the RDW may be a suitable approach for diagnosing IDA in young children [22]. In fact, the adult-derived cut-off value for the RDW of > 13.9% has recently been shown to be suitable when screening for iron deficiency in young children [28]. An increased RDW may even become apparent before manifestation of the anemia (defined as a drop of hemoglobin) itself [29]. The major limitation of the RDW, if used by itself, is the low specificity of 50-60% [25, 30]. It has been proposed to combine the RDW with the MCV (using a cut-off <70 fl) to increase the positive predictive value. However, the MCV (cut-off <70 fl) has likewise a low specificity (53.8%) [25] and hence combining the RDW with the MCV remains problematic if diagnosing IDA. Ferritin (cut off <10 ng/ml), on the other hand, has a specificity close to 100% for IDA or iron deficiency [22, 25]. Hence, we propose that in the case of suspected nutritional iron deficiency a simple laboratory algorithm may be used, combining RDW (cut-off >13.9%) as a sensitive marker and ferritin (cut-off <6 ng/ml) as a marker with high specificity for depleted iron stores.

We acknowledge, however, that this approach also has limitations. In particular, ferritin, being an acute phase protein, may be increased to normal values despite iron-deficiency in the presence of inflammation [31]. Thus, if an infection is clinically suspected, as in the current case, it is required to rule out inflammation using markers like CRP.

In the presented case hemolysis parameters (haptoglobin and bilirubin) were normal and hence a combined anemia (hemolytic and IDA) was ruled out. A possible cause for IDA in small children is prolonged exclusive breastfeeding (EBF) for longer than 6 or 12 months of age [13]. Iron deficiency does not usually become apparent in breastfed infants during the first 6 months as full term infants have sufficient iron stores [32]. However, breast milk is low in iron, and the iron concentration of breast milk declines during the course of lactation [33, 34]. Hence, to prevent iron deficiency during infancy it is recommended to supplement bio-available iron via complementary food starting at age 4–6 months [2]. In the presented case prolonged EBF (>2 years) led to symptomatic IDA. Therefore, despite the contradicting data that mainly considered prolonged EBF associated IDA at earlier time points (6, 9 or 12 months) this case outlines the importance of considering prolonged EBF associated IDA even in older toddlers [13, 14]. Current guidelines addressing the time to terminate EBF, when to commence iron supplementation, and how to screen for IDA in infants and small children need to consider such rare cases of unusually prolonged EBF as well. So even though a recent analysis showed an increased risk for iron deficiency of 4.8% for each month of breastfeeding no significant correlation could be found between total breastfeeding time and iron deficiency anemia [15]. The main variable analyzed in the study by Maguire *et al.* was “total breastfeeding duration”. This, however, combined children with exclusive and non-exclusive breastfeeding and hence the degree and impact of food supplementation remains unaddressed [15]. Also, the analysis found a stronger (but not significant) association between total breastfeeding duration and serum ferritin in children aged <2 years compared to >2 years at follow-up [15]. The crucial question, especially in respect to our case, is whether this is due to the higher proportion of food-supplemented children in the >2 years group. Hence, studies addressing specifically the aspect of exclusive breastfeeding are still lacking.

Furthermore iron supplementation for EBF showed no preventive effect on IDA at 9 months in some cohorts, emphasizing the need for evidence of a causative relationship of EBF and iron deficiency anemia [19]. Recent data revealed that more than (or equal to) 6 breastfeeds per day are associated with insufficient iron intake at 8 months, even if solid supplements are introduced at 6 months of age (41.5% with an iron intake <4.2 mg in the ≥6 breastfeeds/d group) [35]. Our report, adding a case of a 2.5 year old toddler to the literature, indicates that the same holds true for this age group.

The current case suggests for older toddlers a causative link between prolonged EBF and IDA. Iron supplementation was an effective intervention in this case to resolve IDA. Even after such prolonged time of nutritional deficiency, iron supplementation effectively resolved the anemia. At first sight this contradicts recent reports that demonstrated no difference of the effect of iron supplementation on anemia in exclusively breastfed cohorts compared to placebo treated controls [19]. However, it is important to note that in that report iron was supplemented at 4 months of age suggesting that iron is not able to influence hemoglobin synthesis at all stages. There are reports that demonstrated a benefit of iron supplementation, even though the benefit was not sustained [17]. This case shows the therapeutic efficacy of iron supplementation in advanced EBF associated IDA, prompting to raise further discussions on when and how iron supplementation with prolonged breastfeeding should be performed.

Additionally, the current case demonstrates the importance of evaluating the basic nutritional status, that includes anthropometric indicators, dietary information, medical history, behaviors around food consumption, biochemical and clinical indicators (physical signs) and history of medical conditions that potentially affect nourishment [36]. The nutritional assessment may identify some of the established risk factors for iron deficiency anemia such as low birth weight, high cow’s-milk intake, low intake of iron-rich complementary foods, low socioeconomic status, and immigrant status [37]. Similarly, we noted the carious dentition during the physical examination, which has recently been reported to be strongly associated with anemia [38]. Thus, a detailed history and thorough physical examination will provide important hints to diagnosing IDA. Together with the simple but highly indicative combination of low ferritin and increased RDW this enables the quick diagnosis of iron deficiency in infants. A quick and correct diagnosis is important in such cases to prevent further developmental impairment.

## Conclusion

The case demonstrates that unusually prolonged EBF even in older toddlers (>2 years) is a potential cause of IDA. Breastfeeding dependent IDA has recently largely been considered and associated with EBF at 6 or 12 months. Hence, the diagnosis of iron deficiency anemia secondary to unusually prolonged breastfeeding has to be taken into consideration in toddlers, in particular considering the increasingly heterogeneous cultural background of patients. As RBC indices perform weakly in discriminating iron deficiency from thalassemia, we propose a simple combination of ferritin and RDW which in this case enabled a quick diagnosis. An acute infection may be ruled out by CRP. Oral iron supplementation was, even after such prolonged nutritional iron deficiency an effective intervention. Parental counseling on infant feeding practices according to current WHO recommendations [20] and about the risks associated with prolonged breastfeeding are crucial.

### Consent

Written informed consent was obtained from the patient’s legal guardian(s) for publication of this case report and any accompanying images. A copy of the written consent is available for review by the Editor-in-Chief of this journal.

## Abbreviations

AAP: American Academy of Pediatrics
CRP: C-reactive protein
EBF: Exclusive breastfeeding
Hb: Hemoglobin
IDA: Iron deficiency anemia
MCV: Mean corpuscular volume
MCH: Mean corpuscular hemoglobin
RBC: Red blood cells
RDW: Red blood cell distribution width
WHO: World Health Organization.
